# BCL6 promotes the progression of high-grade serous ovarian cancer cells by inhibiting PLAAT4

**DOI:** 10.3389/fphar.2025.1634995

**Published:** 2025-07-24

**Authors:** An Wan, Wei-Dong Zhao, Gang Chen, Cheng Peng, Jin-Hui Tao

**Affiliations:** ^1^ Department of Allergy and Clinical Immunity, The First Affiliated Hospital of University of Science and Technology of China, Division of Life Sciences and Medicine, University of Science and Technology of China, Hefei, China; ^2^ Department of Obstetrics and Gynecology, Division of Life Sciences and Medicine, The First Affiliated Hospital of University of Science and Technology of China, University of Science and Technology of China, Hefei, China; ^3^ Department of Rheumatology and Immunology, Division of Life Sciences and Medicine, The First Affiliated Hospital of University of Science and Technology of China, University of Science and Technology of China, Hefei, China; ^4^ Obstetrics and Gynecology Center, The First Affiliated Hospital of Anhui University of Chinese Medicine, Hefei, Anhui, China

**Keywords:** BCL6, PLAAT4, high-grade serous ovarian cancer, Akt, ovarian cancer

## Abstract

**Background:**

B-cell lymphoma 6 (BCL6) is increasingly recognized as a driver of cancer progression; however, the precise molecular mechanisms by which BCL6 facilitates high-grade serous ovarian cancer (HGSOC) progression remain incompletely understood.

**Methods:**

In this study, immunohistochemical (IHC) staining was used to evaluate the expression of BCL6 and PLAAT4 in HGSOC tissues and normal tissues. Cleavage under targets and tagmentation (CUT&Tag) was combined with RNA sequencing (RNA-seq) analyses to screen and identify the downstream regulatory mechanisms of BCL6. Wound healing assays, plate cloning, EdU, and transwell assays were used to analyze cell proliferation and invasion. The expression of PI3K-AKT, EMT, and proliferation markers were analyzed by immunohistochemistry *in vivo* or by Western blot *in vitro*. *In vivo*, we established a subcutaneous transplantation tumor model and abdominal metastasis model in nude mice to verify the role of BCL6 and PLAAT4 in HGSOC progression.

**Results:**

Clinical analyses revealed that BCL6 expression is significantly elevated in high-grade serous ovarian cancer (HGSOC) tissues compared with that in normal tissues, whereas PLAAT4 expression is reduced. Moreover, high BCL6 and low PLAAT4 expression are associated with poor prognosis in patients with HGSOC. Biological function tests showed that BCL6 contributes to tumor cell proliferation, invasion, and migration, and plays an important role in the progression of HGSOC *in vivo*. Mechanistically, our investigation revealed that BCL6 promotes HGSOC progression by downregulating PLAAT4, thereby influencing the activation of the phosphoinositide 3-kinase (PI3K)/protein kinase B (AKT) signaling pathway.

**Conclusion:**

Collectively, these findings elucidate the pivotal role of the BCL6-PLAAT4-AKT axis in HGSOC progression, establishing a molecular framework for targeting this pathway as a potential therapeutic strategy against HGSOC.

## 1 Introduction

Ovarian cancer is the most lethal gynecologic malignancy, contributing to approximately 207 thousand female deaths worldwide in 2022 (Bray et al., 2024; Zeng et al., 2024). The most common subtype, high-grade serous ovarian cancer (HGSOC), is responsible for more than 80% of all deaths due to ovarian cancer (Xu et al., 2022; Yang et al., 2024). HGSOC mortality has remained unchanged for decades despite advancements in neoadjuvant therapies and surgical approaches (Bast et al., 2019). The 5-year overall survival of patients with HGSOC is estimated to be lower than 40% (Qian et al., 2023). Therefore, there is an urgent need to understand the molecular mechanisms underlying HGSOC progression to identify new therapeutic avenues.

Previous studies have revealed a correlation between elevated expression of BCL6 and poor prognosis, as well as cisplatin resistance, in HGSOC treatment (Zhu et al., 2017; Wang et al., 2020; Shen et al., 2020). Consequently, selective targeting of BCL6 has been proposed as a potential therapeutic strategy for HGSOC (Wu et al., 2024). However, the molecular mechanisms underlying the involvement of BCL6 in HGSOC progression remain unclear.

B-cell lymphoma 6 (BCL6) is a sequence-specific transcriptional repressor belonging to the Bric-a-brac (BTB) family and is characterized by an N-terminal BTB domain. It possesses a trimodular structure consisting of an N-terminal broad-complex, Tramtrack, and BTB domain, a central transcriptional repressor domain 2 (RD2), and a series of C-terminal C2H2 zinc fingers. BCL6 exerts its transcriptional repressive effects by recruiting specific chromatin-modifying corepressor complexes to the lateral groove BTB domain (Miles et al., 2005) and central transcriptional RD2 (Fujita et al., 2004; Mendez et al., 2008). Initially discovered as a critical transcriptional repressor in germinal centers (GCs), BCL6 enables GC B cells to tolerate DNA damage and undergo rapid proliferation by repressing DNA damage-sensing genes and proliferation checkpoint genes, potentially leading to diffuse large B-cell lymphoma. In addition to hematological tumors (Kawabata et al., 2021), elevated BCL6 protein levels have been observed in various solid tumors such as bladder carcinoma, breast cancer, and ovarian cancer (Fernando et al., 2019; Cardenas et al., 2017; De Santis et al., 2022). In these tumors, BCL6 silences a range of target genes in a cell context-dependent manner. Therefore, identification of the key target genes regulated by BCL6 in various tumors should contribute to the understanding of their pathogenesis.

In this study, we reveal a correlation between elevated BCL6 levels and poor prognosis in patients with HGSOC. BCL6 significantly promotes the proliferation, invasion, and migration of HGSOC cells, and this effect is associated with the activation of the PI3K-AKT pathway in HGSOC cells by BCL6. Furthermore, we find that BCL6 activates the PI3K‒AKT pathway through phospholipase A and acyltransferase 4 (PLAAT4) regulation. BCL6 directly binds to the region near the transcription start site (TSS) of PLAAT4, downregulating PLAAT4 expression and thereby promotion AKT phosphorylation. Our study establishes that the BCL6-PLAAT4-AKT axis has potential value in predicting clinical outcomes and is a candidate therapeutic target.

## 2 Materials and methods

### 2.1 Patients and specimens

For this study, 41 HGSOC and 40 fallopian tube (resected for non-ovarian diseases) tissues were obtained from the Department of Pathology, the First Affiliated Hospital of University of Science and Technology of China (USTC), between January 2015 and December 2018. All patients with HGSOC received primary surgery followed by platinum-based chemotherapy. Histopathological reviews of all the samples were conducted by three senior pathologists. The study protocols were approved by the Internal Review and Medical Ethics Committee of the First Affiliated Hospital of USTC (2024ky192), and informed consent was obtained from each participant. To determine the overall survival (OS) of patients with HGSOC, we used the date of initial surgery as the starting point and the date of death or last follow-up as the end point.

### 2.2 Cell lines and cell culture

Human ovarian tumor cell lines SKOV3 and COV504 were purchased from the American Type Culture Collection (ATCC) and European Collection of Authenticated Cell Cultures (ECACC), respectively. Human embryonic kidney (HEK) 293T cells were purchased from the Cell Bank of the Chinese Academy of Science (Shanghai, China). SKOV3, COV504, and 293T cells were cultured in DMEM (Gibco, United States) supplemented with 10% fetal bovine serum (FBS) (Gibco, United States). SKOV3 and COV504 cells were stably transfected with either BCL6 or PLAAT4 to obtain BCL6-overexpressing cells or PLAAT4-overexpressing cells. After transfection, the cells were maintained in medium supplemented with a lethal dose of G418 or puromycin (2 mg/mL G418 for BCL6-overexpressing cells and 5 μg/mL puromycin for PLAAT4-overexpressing cells). The selected clones were maintained in DMEM with a half-lethal dose of G418 or puromycin for each cell line. SKOV3 and COV504 cells were transfected with single-guide RNA (sgRNA) for specific sequences in BCL6 (sg-BCL6: 5′-CGA​CCA​AGC​TCA​GTG​CCA​GT-3′) and then selected in culture medium supplemented with 5 μg/mL puromycin. A single colony of BCL6-knockout cells was picked and expanded in medium supplemented with 2 μg/mL puromycin. For transient knockdown, SKOV3 and COV504 cells were transfected with appropriate amounts of negative control or siRNA for PLAAT4 (Hanbio Tech, Shanghai, China) via Lipofectamine RNAiMAX (Life Technologies) in accordance with the manufacturer’s instructions.

### 2.3 Cleavage under targets and tagmentation (CUT&Tag)

A total of 100,000 BCL6-overexpressing SKOV3 cells were used. The CUT&Tag assay and subsequent DNA library construction were performed in accordance with the manufacturer’s instructions for the HyperactiveTM *In Situ* ChIP Library Prep Kit for Illumina (TD902, Vazyme Biotech, China) (Kaya-Okur et al., 2019). An anti-BCL6 rabbit antibody (CST, #14895) was used as the primary antibody. Hyperactive pA-Tn5 transposase was used to precisely cut off the DNA sequence near the target protein under antibody guidance to obtain DNA fragments that could be ligated with P5 and P7 adaptors. The DNA library was evaluated with an Agilent 2100 Bioanalyzer. Sequencing was performed on an Illumina NovaSeq 6000 platform. Then, 150-bp reads were generated for subsequent analysis via fastp, Bowtie2, SEACR, chipseeker, MEME, and DREME software.

### 2.4 RNA extraction and mRNA sequencing

Total RNA was extracted from tumor cells with TRIzol (Life Technologies). RNA purity and integrity were evaluated (RIN > 6.0 and a 28S:18S ratio >1.5) with a NanoDrop spectrophotometer and an Agilent 2100 Bioanalyzer. Sequencing libraries for transcriptomic analysis were generated by OE Biotech Co., Ltd. (Shanghai, China) via the VAHTS Universal V6 RNA-seq Library Prep Kit. Sequencing was performed on an Illumina NovaSeq 6000 platform. Then, 150-bp reads were generated for subsequent analysis via fastp, HISAT2, HTSeq-count, R (v 3.2.0), and gene set enrichment analysis (GSEA) software.

### 2.5 Luciferase assay

For the dual-luciferase reporter assay, the *PLAAT4* promoter (2,000 bp) and mutant *PLAAT4* promoter (deleted sequence CATGGTGA) were inserted into the PGL4.23 vector to generate the *PLAAT4*-wild type (WT) reporter vector and *PLAAT4*-mutant type (MUT) reporter vector, respectively. The reporter vector was subsequently cotransformed via Lipofectamine 2000 into 293T cells, together with the negative control vector (pCMV-NC) or the effector vector (pCMV-BCL6). After the 293T cells were cultured at 37°C for 48 h, the Dual-Luciferase Assay System (Promega) was used to determine the ratio of firefly luciferase activity to *Renilla* luciferase activity.

### 2.6 Reverse transcription-quantitative PCR (RT-qPCR)

Total RNA from SKOV3 and COV504 cells was reverse-transcribed into first-strand DNA using the PrimeScript RT Reagent Kit (TaKaRa, Dalian, China) in accordance with the manufacturer’s instructions. The expression levels of *BCL6* and *PLAAT4* were detected by quantitative PCR with SYBR Premix Ex Taq (Takara, Dalian, China). *GAPDH* expression was used as the internal reference, and the following primer pairs were used for qPCR:

Human-*GAPDH*-F GTG​AAG​GTC​GGA​GTC​AAC​G.

Human-*GAPDH*-R TGA​GGT​CAA​TGA​AGG​GGT​C.

Human-*BCL6*-F GGA​GTC​GAG​ACA​TCT​TGA​CTG​A.

Human-*BCL6*-R ATG​AGG​ACC​GTT​TTA​TGG​GCT.

Human-*PLAAT4*-F GAG​ATT​TTC​CGC​CTT​GGC​TAT.

Human-*PLAAT4*-R CCG​GGG​TAC​TCA​CTT​GGA​G.

### 2.7 Western blotting

Cellular protein was extracted from SKOV3 and COV504 cells using RIPA buffer supplemented with protease and phosphatase inhibitors. Then, 7.5%–12.5% SDS‒PAGE was performed to separate the cellular proteins, which were subsequently transferred onto a nitrocellulose (NC) membrane in an ice bath. The NC membrane was blocked in 5% skim milk powder at 25°C for 2 h and incubated with primary antibodies overnight at 4°C, followed by 1 h of incubation with secondary antibodies at room temperature. Luminescence was detected with an electrochemiluminescence (ECL) reagent kit. Primary antibodies and secondary antibodies were purchased from the following sources: antibodies against p-Akt (Sec473, #4060), Akt (#4691), BCL6 (#14895), E-cadherin (#3195), vimentin (#5741), MMP-1 (#54376), cyclin D1 (#55506), and β-actin (#3700) were purchased from Cell Signaling Technology. An antibody against PLAAT4/TIG3 (ab96468) was purchased from Abcam. Secondary antibodies, including HRP-labeled goat anti-mouse IgG and HRP-labeled goat anti-rabbit IgG, were purchased from Proteintech. Each experiment was performed in triplicate.

### 2.8 Colony formation assay

Stably transfected SKOV3 and COV504 cells (1 × 10^3^ cells/well) were seeded in six-well plates and incubated in DMEM with 10% FBS at 37°C in 5% CO_2_ for at least 10 days. The cells were then fixed with paraformaldehyde (4%) for 30 min at 25°C and stained with 0.2% crystal violet methanolic solution for 1 h at 25°C. The number of clones was counted by eye. Triplicate biological replicates were performed.

### 2.9 Wound-healing assay

The cells for the wound-healing assay were cultured in 6-well plates in DMEM supplemented with 10% FBS until full confluence was reached. A wound was then created with a sterile tip. After gently washing with PBS to remove the shed cells, the cells were cultured in 2 mL of medium supplemented with 5% FBS at 37°C in 5% CO_2_ for 2 days. The same wound location was imaged at 0 h and 48 h under a light microscope (Olympus, Tokyo, Japan). The assay was repeated independently at least three times. Triplicate biological replicates were performed.

### 2.10 Invasion assays

Transwell assays were performed to evaluate the invasion ability of the cells in transwell chambers (Corning 3422, 8 μm pore size) with Matrigel (BD Bioscience, United States). In detail, 4 × 10^4^ SKOV3 or COV504 cells were seeded into the upper chamber, which contained 200 μL of DMEM without FBS. Meanwhile, DMEM (500 μL) containing 10% FBS was added to the bottom of a 24-well plate. The cells were incubated at 37°C for 48 h, and the cells remaining at the upper surface of the filter membrane were scraped with a cotton swab. The cells that passed through the filter membrane were fixed with paraformaldehyde (4%) for 30 min at room temperature and stained with 0.2% crystal violet methanolic solution for 1 h at room temperature. The membrane was imaged under a light microscope (Olympus, Tokyo, Japan). Triplicate biological replicates were performed.

### 2.11 5-Ethynyl-2-deoxyuridine (EdU) proliferation assay

SKOV3 or COV504 cells were seeded in a 24-well plate. After incubation with the EdU buffer and cell medium for 2 h, cell proliferation was measured with a Cell Proliferation Assay Kit (BeyoClickTM EdU-488). In brief, the cells were fixed with 4% paraformaldehyde and permeabilized with 0.5% Triton X-100. The cells were subsequently stained with Hoechst and visualized via fluorescence microscopy (Olympus, Japan). Triplicate biological replicates were performed.

### 2.12 Animal tumor model

The animal experiments were approved by the Animal Care and Use Committee of the First Affiliated Hospital of USTC [2024-N(A)-081]. Six-week-old female athymic nude mice were obtained from GemPharmatech Co., Ltd., and randomly divided into different groups. A total of 5 × 10^6^ SKOV3 cells were subcutaneously injected into the nude mice. After 4 weeks, tumors were removed and weighed. A total of 5 × 10^6^ SKOV3-Luc bearing cells with either stable expression of pCMV-BCL6 or pCMV-PLAAT4 were intraperitoneally injected into the nude mice. After 5 weeks, D-luciferin solution was injected intraperitoneally, and the mice were placed in the imaging chamber of the IVIS Lumina II system.

### 2.13 Immunohistochemistry

Tissue samples were deparaffinized in xylene and rehydrated in ethanol. After heat-induced epitope retrieval, the sections were blocked with 3% hydrogen peroxide at room temperature for 30 min and subsequently stained with antibodies against BCL6 (Abcam, 1:200, ab172610) or PLAAT4/RARRES3 (Proteintech, 1:200, 12065-1-AP) overnight at 4°C. The next day, the sections were incubated with a secondary antibody, and the color reaction was then performed using a DAB Horseradish Peroxidase Color Development Kit. Brownish granules in the cytoplasm confirmed the presence of PLAAT4 antibodies and in the nucleus confirmed the presence of BCL6 antibodies. After initial low-power screening, scoring was performed using 200× magnification power to determine the percentage of stained tumor cells and staining intensity.

The expression of BCL6 or PLAAT4 was calculated on the basis of the proportion of positively stained cells (0: 0%–4% tumor cells stained, 1: 5%–25%, 2: 26%–50%, 3: 51%–75%, 4: 76%–100%) and the staining intensity score (0: no staining, 1: weak staining, 2: moderate staining, and 3: strong staining). The immunohistochemistry score was obtained by multiplying these two numbers (−: 0−−2, +: 3−−4, ++: 5−−8, +++: 9−−12). Three senior pathologists scored each tissue slice independently to reduce errors.

### 2.14 Statistical analysis

Data were compared by unpaired two-tailed Student’s t tests (for continuous characteristics) or χ^2^ test (for categorical characteristics). Experimental data were analyzed by SPSS 26.0 software. The relationship between BCL6 and PLAAT4 expression in HGSOC tissue was analyzed via Spearman’s correlation coefficient. Survival analysis was performed via the Kaplan-Meier method. A two-sided P value <0.05 was considered to indicate statistical significance.

## 3 Results

### 3.1 BCL6 induces malignant behaviors in HGSOC cells

**FIGURE 1:**
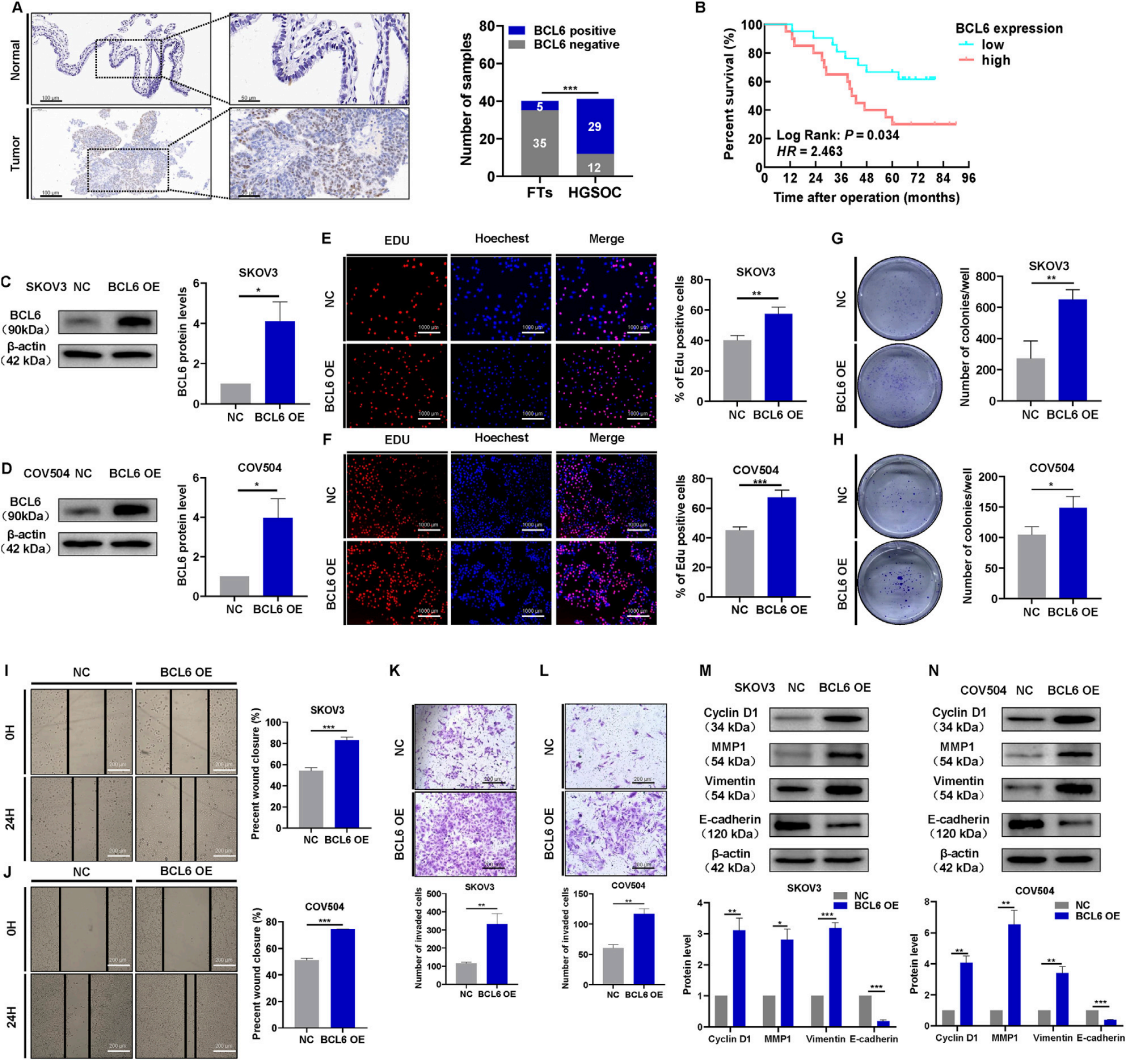
BCL6 induces malignant behaviors in HGSOC cells. **(A)** Representative images of BCL6 protein staining by IHC in normal fallopian tube and HGSOC tissues. **(B)** Kaplan-Meier analysis of overall survival in 41 patients with HGSOC stratified by BCL6 expression levels. **(C,D)** Western blotting analysis of the HGSOC cell lines SKOV3 **(C)** and COV504 **(D)** after transfection with pCMV-BCL6-Neo plasmid to overexpress BCL6. **(E)** Assessment of cell proliferation in the cells from **(C)**, measured by EdU assays (scale bar = 1,000 µm). **(F)** Assessment of cell proliferation in the cells from **(D)**, measured by EdU assays (scale bar = 1,000 µm). **(G)** Assessment of cell colony formation ability in the cells from **(C)**, measured by colony formation assays. **(H)** Assessment of cell colony formation ability in the cells from **(D)**, measured by colony formation assays. **(I)** Assessment of migration in the cells from **(C)**, measured by wound healing assays (scale bar = 200 µm). **(J)** Assessment of migration in the cells from **(D)**, measured by wound healing assays (scale bar = 200 µm). **(K)** Assessment of invasion in the cells from **(C)**, measured by transwell assays (scale bar = 200 µm). **(L)** Assessment of invasiveness of cells from **(D)**, measured by transwell assays (scale bar = 200 µm). **(M,N)** Western blotting analysis of cyclin D1, vimentin, E-cadherin, and MMP1 in total cell lysates after overexpression of BCL6 in SKOV3 and COV504 cells. The data are presented as the means ± SDs of n = 3 independent biological experiments; two-tailed Student’s t-test, *P < 0.05, **P < 0.01, ***P < 0.001, ns, not significant.

First, we detected BCL6 protein expression in HGSOC tissues and normal fallopian tube tissues. Of the 40 normal fallopian tube tissues samples, only 12.5% (n = 5) samples showed positive expression of BCL6. BCL6 expression was upregulated in 80.49% (n = 29) HGSOC samples, and weak BCL6 cytoplasmic staining was observed in some samples (Figure 1A). Positive expression of BCL6 varied significantly between HGSOC tissues and normal fallopian tube tissues. Next, we examined the associations between BCL6 expression and patient clinicopathological data. BCL6 expression was associated with advanced tumor stage and a higher incidence of metastasis to the lymph nodes, omentum, and mesentery (Table 1). Compared with patients with other FIGO stages of HGSOC, more patients with FIGO stage III/IV HGSOC had high BCL6 expression in tumor cells. In addition, patients with HGSOC who exhibited high BCL6 expression in primary tumors had significantly poorer OS (Figure 1B), indicating that BCL6 expression influences patient outcomes. To investigate the biological function of BCL6 in HGSOC cells, BCL6 expression was stably knocked out using sgRNA (Supplementary Figures S1A,B), and cell lines with stable overexpression of BCL6 were established using the pCMV-BCL6-Neo plasmid (Figures 1C,D). EdU and colony formation assays revealed that knockout of BCL6 suppressed cell proliferation and limited the colony formation ability of SKOV3 and COV504 cells (Supplementary Figures S1C–F), whereas ectopic expression of BCL6 resulted in the opposite trend (Figures 1E–H). Additionally, the effects of BCL6 on the invasive and migratory abilities of SKOV3 and COV504 cells were further examined via transwell assays with Matrigel and wound healing assays. As shown in Supplementary Figures S1G–J, BCL6 knockout significantly reduced the migratory and invasive capacity of SKOV3 and COV504 cells, whereas BCL6 overexpression had the opposite effect (Figures 1I–L). Indeed, examination of the key markers of proliferation, migration, and invasion via Western blotting revealed changes consistent with those observed via cell-based functional assays. The overexpression of BCL6 reduced the expression of E-cadherin and increased the expression of cyclin D1, vimentin, and MMP1 in SKOV3 and COV504 cells (Figures 1M,N). In contrast, the silencing of BCL6 in SKOV3 and COV504 cells had the opposite effects, with increases in E-cadherin and decreases in cyclin D1, vimentin, and MMP1 (Supplementary Figures S1K,L). To further explore the role of BCL6 in the development of HGSOC, we injected SKOV3 cells transfected with sg-BCL6 or sg-NC subcutaneously into nude mice to establish xenografts. Continuous measurement of tumor weight revealed that xenografts derived from sg-BCL6 cells grew much slower than those derived from sg-NC cells (Supplementary Figure S1M). Collectively, these data suggest that BCL6 promotes malignant behaviors in HGSOC cells.

**TABLE 1:** Association between the expression levels of BCL6 and the clinical parameters of patients with HGSOC [*n* (%)].

Characteristics	Overall	BCL6 expression		χ^2^	*P* value
		Low (*n* = 21)	High (*n* = 20)		
Age (years)				0.620	0.431
<50	12 (29.27)	5 (23.81)	7 (35.00)		
≥50	29 (70.73)	16 (76.19)	13 (65.00)		
CA125 (U/mL)				0.067	0.796
<1,000	30 (73.17)	15 (71.43)	15 (75.00)		
≥1,000	11 (26.83)	6 (28.57)	5 (25.00)		
FIGO stage				6.131^a^	0.013
Ⅰ + Ⅱ	15 (36.59)	12 (57.14)	3 (15.00)		
Ⅲ + Ⅳ	26 (63.41)	9 (42.86)	17 (85.00)		
Involvement					
Lymph nodes				7.037	0.008
Yes	20 (48.78)	6 (28.57)	14 (70.00)		
No	21 (51.22)	15 (71.43)	6 (30.00)		
Omentum^a^				4.193	0.041
Yes	22 (53.66)	8 (38.10)	14 (70.00)		
No	19 (46.34)	13 (61.90)	6 (30.00)		
Mesentery^a^				5.467	0.019
Yes	19 (46.34)	6 (28.57)	13 (65.00)		
No	22 (53.66)	15 (71.43)	7 (35.00)		
Other sites				3.029	0.082
Yes	15 (36.59)	5 (23.81)	10 (50.00)		
No	26 (63.41)	16 (76.19)	10 (50.00)		

^a^ Adjusted chi-square test; other sites, including the peritoneum, diaphragm and liver.

### 3.2 BCL6 promotes the proliferation, migration, and invasion of HGSOC cells via the PI3K/AKT pathway

**FIGURE 2:**
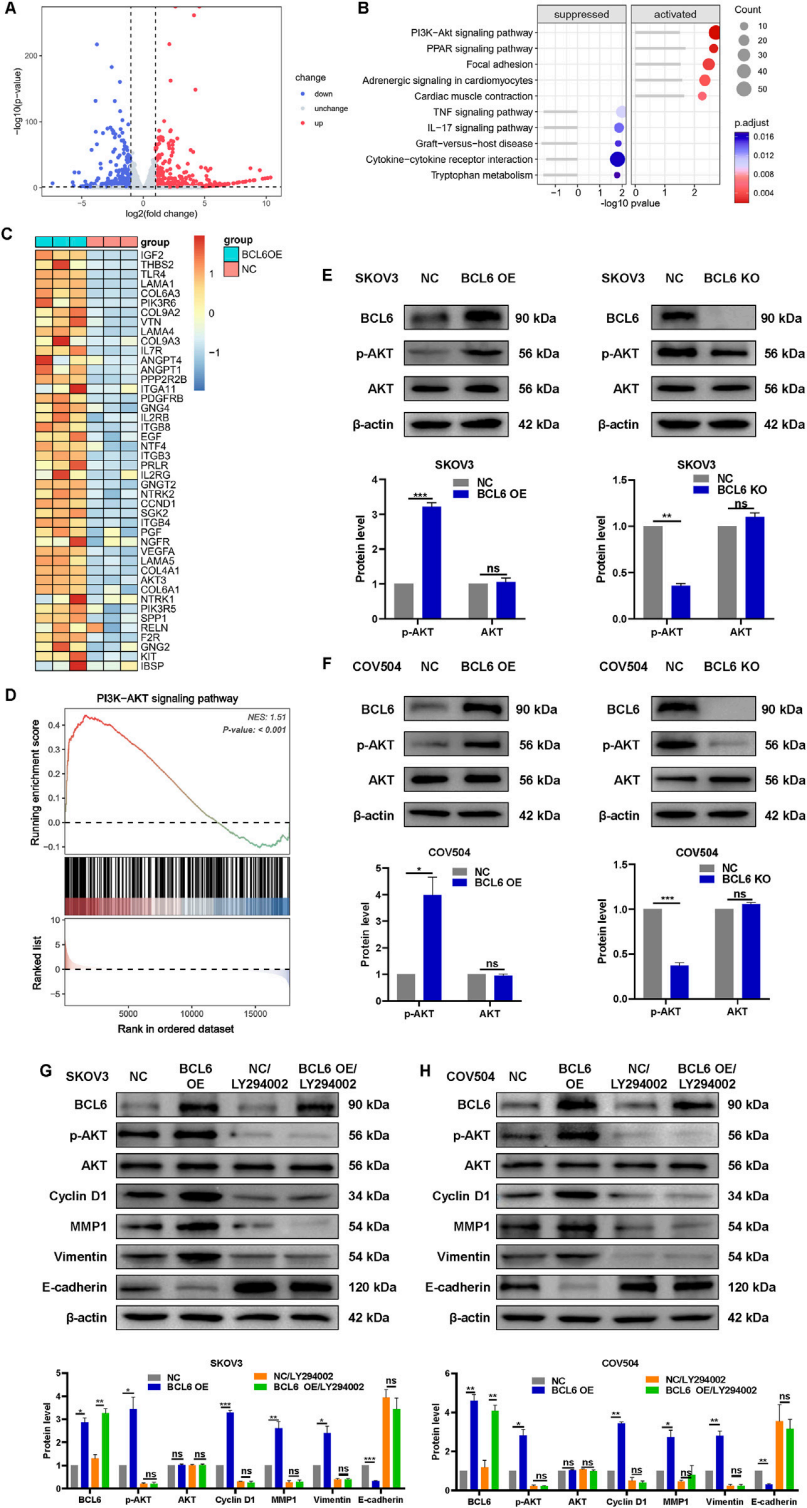
BCL6 promotes the proliferation, migration, and invasion of HGSOC cells via the PI3K/AKT pathway. **(A)** Volcano plots of the differentially expressed genes, with 419 upregulated genes and 457 downregulated genes, in SKOV3 cells transfected with pCMV-BCL6 relative to SKOV3 cells transfected with pCMV-NC via RNA-sequencing (RNA-seq) analysis. **(B)** KEGG analysis of the differentially expressed genes in **(A)**. **(C)** Heatmap summarizing genes differentially expressed in **(A)**. **(D)** Gene set enrichment analysis (GSEA) output images of the PI3K-AKT pathway. **(E,F)** Western blotting analysis of AKT and p-AKT in total cell lysates after overexpression of BCL6 or after knockout of BCL6 in SKOV3 and COV504 cells. **(G,H**) Relative expression levels of E-cadherin, vimentin, MMP1, cyclin D1, AKT, p-AKT, and BCL6 in the indicated cells treated with or without LY294002. The data are presented as the means ± SDs of n = 3 independent biological experiments; two-tailed Student’s t-test; *P < 0.05, **P < 0.01, ***P < 0.001, ns, not significant.

To further explore the potential molecular mechanism by which BCL6 induces malignant behavior in HGSOC cells, mRNA sequencing was performed on three pairs of BCL6-overexpressing and normal control SKOV3 cells. Compared with the NC group, 876 differentially expressed genes (DEGs) were identified in SKOV3 cells transfected with pCMV-BCL6 (Figure 2A). KEGG pathway analysis was carried out to annotate the functions of these 876 genes. The PI3K-AKT pathway, which plays an important role in the occurrence and progression of tumors (He et al., 2021), had the highest score in the enrichment analysis (Figure 2B). GSEA further revealed that the PI3K-AKT signaling pathway was significantly activated by BCL6 (Figures 2C,D). The protein levels of key components of the PI3K-AKT signaling pathway were confirmed by Western blotting. BCL6 knockout significantly reduced the protein level of p-AKT but did not significantly affect the total AKT level in SKOV3 or COV504 cells. In contrast, BCL6 overexpression had the opposite effect (Figures 2E,F). To determine whether activation of the PI3K‒AKT pathway is essential for the role of BCL6 in promoting malignant behavior, we treated BCL6-overexpressing SKOV3 or COV504 cells with LY294002 (Jiang et al., 2010). We found that the expression levels of cyclin D1, E-cadherin, vimentin, and MMP1 in LY294002-treated cells were significantly lower than those in BCL6-overexpressing cells (Figures 2G,H). Western blotting revealed that the BCL6-mediated increase in cell proliferation, migration, and invasion was abolished by LY294002.

### 3.3 BCL6 activates the PI3K-AKT signaling pathway by negatively regulating PLAAT4

**FIGURE 3:**
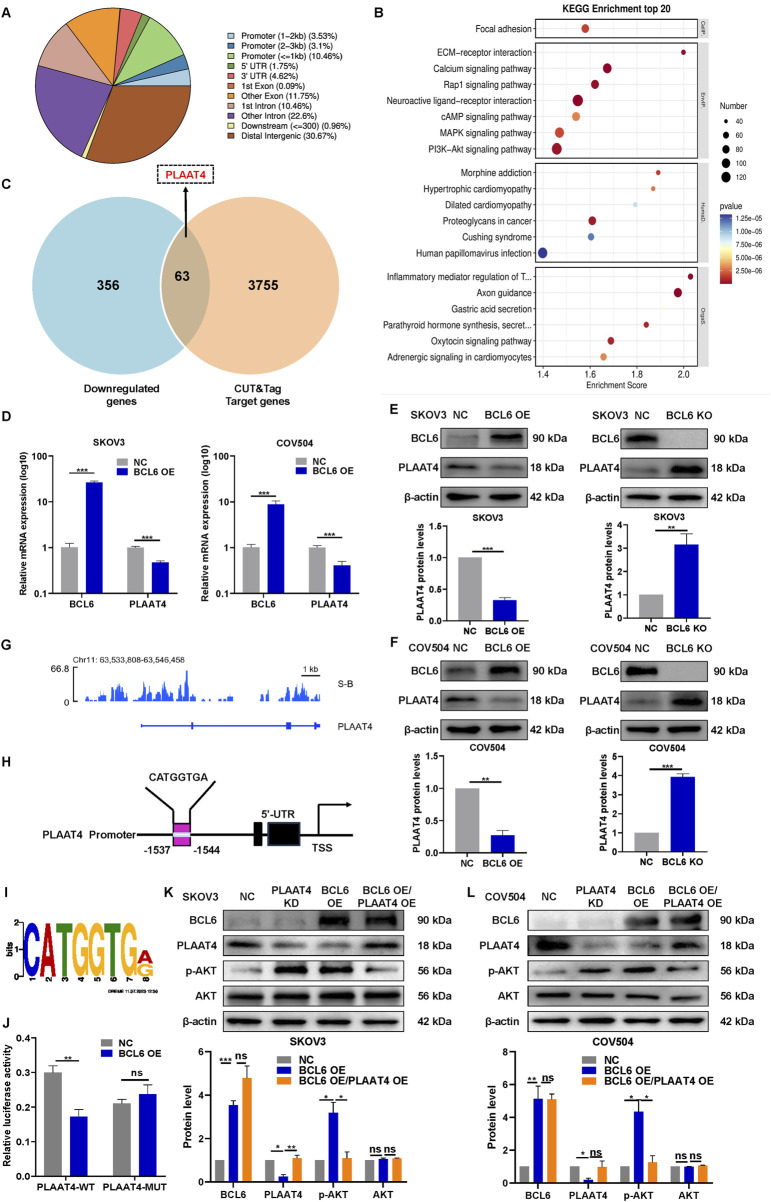
BCL6 activates the PI3K‒AKT signaling pathway by negatively regulating *PLAAT4*. **(A)** Genomic distribution of CUT&Tag peaks in SKOV3 cells. **(B)** KEGG analysis of the peak-related genes in **(A)**. **(C)** Venn diagram of overlapping genes between sets of genes downregulated through RNA-seq and the target gene set identified by CUT&Tag. **(D)** qRT‒PCR revealed that SKOV3 and COV504 cells overexpressing BCL6 presented decreased *PLAAT4* mRNA expression. **(E,F)** Western blotting revealed that SKOV3 and COV504 cells overexpressing BCL6 presented decreased PLAAT4 protein expression, whereas BCL6 knockout resulted in the opposite trend. **(G)** BCL6 peak enrichment in the promoter region of the *PLAAT4* gene according to CUT&Tag analysis. **(H)** Sequence logo of a potential BCL6-binding site in CUT&Tag. **(I)** A diagram of mutant sites in the *PLAAT4* sequence. **(J)** Dual-luciferase reporter assay with the promoter of PLAAT4 and the mutated promoter of PLAAT4 in BCL6-overexpressing SKOV3 cells. **(K,L)** Immunoblot analysis of AKT, p-AKT, BCL6, and PLAAT4 in BCL6-overexpressing SKOV3 and COV504 cells transfected with or without PLAAT4. The data are presented as the means ± SDs of n = 3 independent biological experiments; two-tailed Student’s t-test; *P < 0.05, **P < 0.01, ***P < 0.001, ns, not significant.

Given BCL6 plays a critical role in regulating various cellular processes as a major inhibitor of transcription, we explored the potential downstream target genes associated with the PI3K-AKT pathway by combining RNA-seq and CUT&Tag. First, RNA-seq analysis revealed that 419 genes were downregulated in SKOV3 cells transfected with pCMV-BCL6 (|fold change| ≥ 2, *P* < 0.05). We subsequently studied the genome-wide target sites of BCL6 via the CUT&Tag analysis approach. We identified 26,640 peaks associated with genome binding; their distribution is shown in Figure 3A. KEGG analysis of the peak-related genes revealed that the BCL6 target DEGs are involved in the PI3K-AKT pathway (Figure 3B). This finding was consistent with the finding from RNA-seq analysis that BCL6 activates the PI3K-AKT pathway. Next, we performed RNA-seq together with CUT&Tag analysis to investigate the overlapping gene sets among the DEGs after BCL6 overexpression. We found that 63 downregulated genes were included in the set of BCL6 target genes (Figure 3C). Interestingly, the expression of PLAAT4, a tumor suppressor associated with the PI3K-AKT pathway (Ou et al., 2008; Tsai et al., 2006; Tsai et al., 2009), was negatively correlated with the expression of BCL6 (Figures 3D–F). More importantly, BCL6 peaks were enriched in the *PLAAT4* promoter region (Figure 3G). We also identified the motif sequence (CATGGTGA) as a potential binding site in the *PLAAT4* promoter with the highest relative score in the CUT&Tag analysis (Figures 3H,I). On the basis of this potential binding site, a luciferase reporter assay was conducted in 293T cells cotransfected with BCL6 plasmids. Transfection with the *PLAAT4* promoter-controlled luciferase expression plasmids resulted in a marked reduction in luciferase expression; however, this change did not occur after deleting the sequence (CATGGTGA) with the highest relative score at the *PLAAT4* promoter (Figure 3J). These findings indicate that BCL6 downregulates the expression of *PLAAT4* by inhibiting its transcription. To determine whether the expression of *PLAAT4* is essential for BCL6-mediated activation of the PI3K/AKT pathway, we conducted rescue experiments. Restoration of PLAAT4 expression almost completely suppressed the PI3K/AKT pathway in BCL6-overexpressing SKOV3 and COV504 cells (Figures 3K,L).

### 3.4 PLAAT4 suppresses the malignant behaviors of HGSOC cells

**FIGURE 4:**
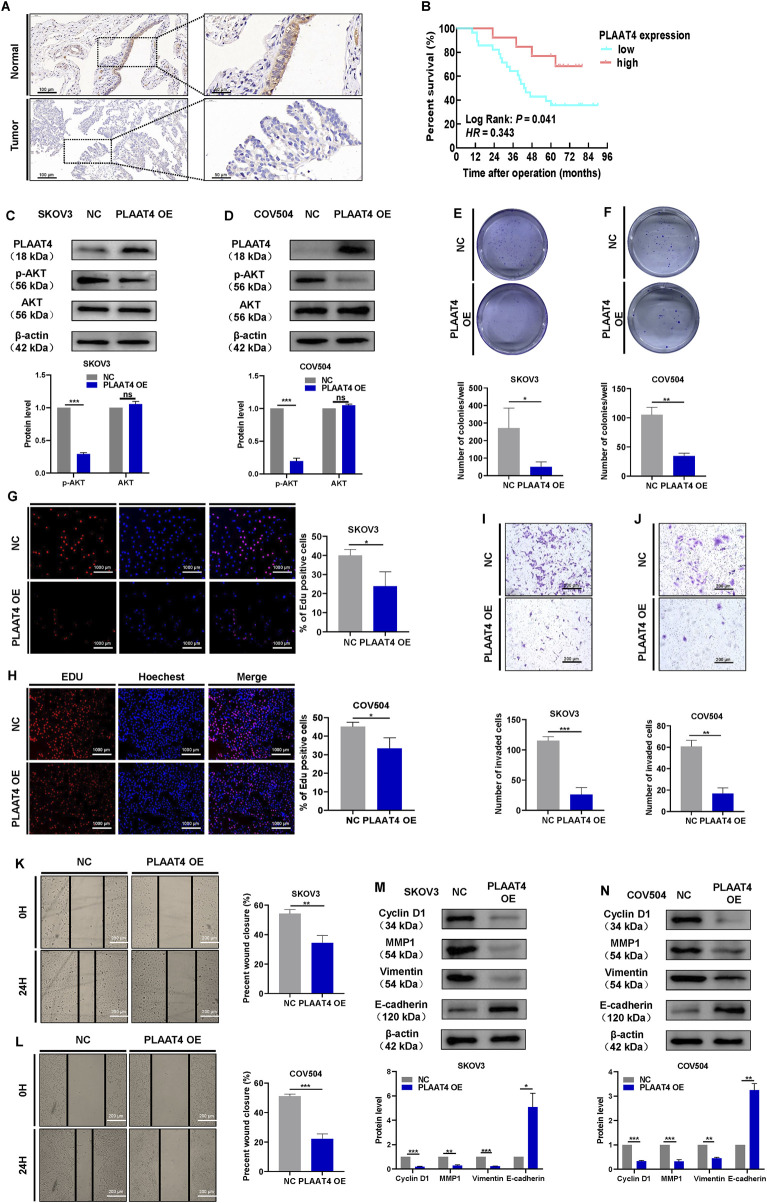
PLAAT4 suppresses malignant behaviors in HGSOC cells. **(A)** Representative images of PLAAT4 protein staining by IHC in normal fallopian tube and HGSOC tissues. **(B)** Kaplan-Meier analysis of overall survival in 41 HGSOC patients stratified by PLAAT4 expression. **(C,D)** Western blot analysis of AKT and p-AKT in total cell lysates after PLAAT4 was overexpressed in SKOV3 and COV504 cells. **(E)** Assessment of the colony-forming ability of SKOV3 cells via colony formation assays. **(F)** Assessment of the colony-forming ability of COV504 via colony formation assays. **(G)** Assessment of proliferation in SKOV3 cells via EdU incorporation assays (scale bar = 1,000 µm). **(H)** Assessment of COV504 proliferation via EdU assays (scale bar = 1,000 µm). **(I)** Assessment of the invasiveness of SKOV3 cells via transwell assays (scale bar = 200 µm). **(J)** Assessment of the invasiveness of COV504 cells via transwell assays (scale bar = 200 µm). **(K)** Assessment of migration in SKOV3 cells via wound-healing assays (scale bar = 200 µm). **(L)** Assessment of COV504 cell migration in wound-healing assays (scale bar = 200 µm). **(M,N)** Western blot analysis of cyclin D1, vimentin, E-cadherin, and MMP1 in total cell lysates after overexpression of PLAAT4 in SKOV3 and COV504 cells.

Firstly, we detected PLAAT4 protein expression in HGSOC tissues and normal fallopian tube tissues. PLAAT4 was expressed in the cell membrane and cytoplasm of the tubal epithelium. Of the 40 normal fallopian tube tissues samples, 100% (n = 40) samples showed positive expression of PLAAT4. PLAAT4 expression was expressed in the cytoplasm of tumor cells (positive rate: 65.85%) at a lower level than in normal fallopian tube tissues (Figure 4A). Next, we examined the association between PLAAT4 expression and patient clinicopathological data and found that low PLAAT4 expression was associated with advanced tumor stage (Table 2). Compared with those with other FIGO stages of HGSOC, more patients with FIGO stage III/IV HGSOC had lower PLAAT4 expression in tumor cells. In addition, patients with HGSOC who presented with lower PLAAT4 expression in primary tumors had significantly poorer OS outcomes (Figure 4B), indicating that PLAAT4 is a key tumor suppressor in HGSOC. Although PLAAT4 was found to be directly regulated by BCL6, little is known about the biological function of PLAAT4 in HGSOC cells. To investigate the role of PLAAT4 in HGSOC cells, PLAAT4 was depleted using siRNA targeting its back-splicing junction sites, and PLAAT4 was stably overexpressed using the pCMV-PLAAT4-puro plasmid. The overexpression of PLAAT4 inactivated the PI3K-AKT signaling pathway (Figures 4C,D). EdU and colony formation assays revealed that ectopic expression of PLAAT4 suppressed cell proliferation and limited the colony formation ability of SKOV3 and COV504 cells (Figures 4E–H). EdU assays revealed that SKOV3 and COV504 cell proliferation was significantly increased after PLAAT4 depletion (Supplementary Figures S2C,D). Additionally, the effects of PLAAT4 on the invasive and migratory abilities of SKOV3 and COV504 cells were further examined via transwell assays with Matrigel and wound healing assays. As shown in Figures 4I–L, PLAAT4 overexpression significantly reduced the migratory and invasive capacity of SKOV3 and COV504 cells, whereas PLAAT4 knockdown had the opposite effect (Supplementary Figures S2E–H). Indeed, examination of the key markers of proliferation, migration, and invasion by Western blotting revealed changes consistent with those revealed by cell-based functional assays. Overexpression of PLAAT4 increased the expression of E-cadherin and reduced the expression of cyclin D1, vimentin, and MMP1 in SKOV3 and COV504 cells (Figures 4M,N). In contrast, the silencing of PLAAT4 in SKOV3 and COV504 cells had the opposite effects, with decreases in E-cadherin and increases in cyclin D1, vimentin, and MMP1 observed (Supplementary Figures S2I,J). Measurement of tumor weights showed that xenografts developed from pCMV-PLAAT4 cells grew much slower than those derived from pCMV-NC cells (Supplementary Figure S2M). Collectively, the above data suggest that PLAAT4 plays a tumor-suppressive role in HGSOC cells.

**TABLE 2:** Association between the expression levels of PLAAT4 and the clinical parameters of patients with HGSOC [n (%)].

Characteristics	Overall	PLAAT4 expression		χ^2^	*P* value
		Low (*n* = 28)	High (*n* = 13)		
Age (years)				0.000	1.000
<50	12 (29.27)	8 (28.57)	4 (30.77)		
≥50	29 (70.73)	20 (71.43)	9 (69.23)		
CA125 (U/mL)				1.312	0.252
<1,000	30 (73.17)	22 (78.57)	8 (61.54)		
≥1,000	11 (26.83)	6 (21.43)	5 (38.46)		
FIGO stage				5.109	0.024
Ⅰ + Ⅱ	15 (36.59)	7 (25.00)	8 (61.54)		
Ⅲ + Ⅳ	26 (63.41)	21 (75.00)	5 (38.46)		
Involvement					
Lymph nodes				1.529	0.216
Yes	20 (48.78)	16 (57.14)	4 (30.77)		
No	21 (51.22)	12 (42.86)	9 (69.23)		
Omentum^a^				1.768	0.184
Yes	22 (53.66)	17 (60.71)	5 (38.46)		
No	19 (46.34)	11 (39.29)	8 (61.54)		
Mesentery^a^				2.887	0.089
Yes	19 (46.34)	16 (57.14)	3 (23.08)		
No	22 (53.66)	12 (42.86)	10 (76.92)		
Other sites				2.471	0.116
Yes	15 (36.59)	13 (46.43)	2 (15.38)		
No	26 (63.41)	15 (53.57)	11 (84.62)		

^a^ Adjusted chi-square test; other sites, including the peritoneum, diaphragm and liver.

### 3.5 BCL6 induces HGSOC cell malignant behaviors, which are mainly dependent on the tumor suppressor protein PLAAT4

**FIGURE 5:**
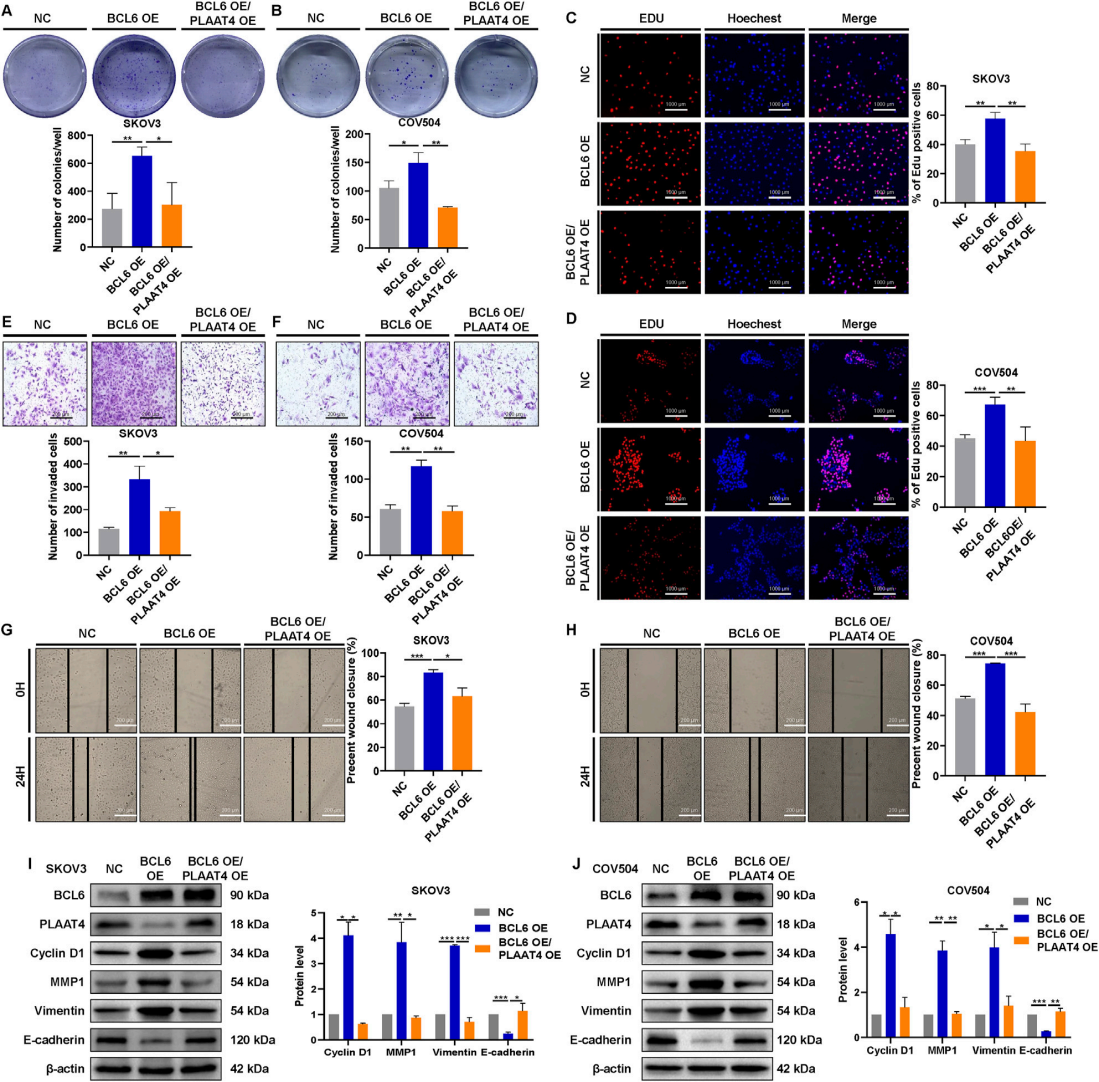
BCL6 induces HGSOC cell malignant behaviors via the tumor suppressor protein PLAAT4. **(A,B)** Colony formation of BCL6-overexpressing SKOV3 and COV504 cells, with or without PLAAT4 re-expression, was measured via colony formation assays. **(C,D)** Proliferation of BCL6-overexpressing SKOV3 and COV504 cells, with or without PLAAT4 re-expression, was measured via EdU assays. **(E–H)** The migration and invasion of BCL6-overexpressing SKOV3 and COV504 cells, with or without PLAAT4 re-expression, were analyzed via transwell and wound healing assays. **(I,J)** Western blot analysis of BCL6, PLAAT4, cyclin D1, vimentin, E-cadherin, and MMP1 in BCL6-overexpressing SKOV3 and COV504 cells with or without PLAAT4 re-expression. The data are presented as the means ± SDs of n = 3 independent biological experiments; two-tailed Student’s t*-*test; *P < 0.05, **P < 0.01, ***P < 0.001, ns, not significant.

Re-expression of PLAAT4 reversed the activation of the PI3K/AKT pathway in BCL6-overexpressing SKOV3 or COV504 cells, and PLAAT4 was found to play a tumor suppressor role in HGSOC cells. Therefore, we next explored whether and to what extent, PLAAT4 could restore the malignant capacity of BCL6-overexpressing SKOV3 and COV504 cells. We stably transfected cells overexpressing BCL6 with a plasmid expressing PLAAT4 or a control plasmid. Re-expression of PLAAT4 partially restored the cell growth and proliferation enhanced by BCL6 overexpression, as demonstrated by colony formation and EdU assays (Figures 5A–D). In addition, transwell and wound-healing assays revealed that the migratory and invasive abilities of BCL6-overexpressing SKOV3 and COV504 cells were significantly suppressed by PLAAT4 re-expression (Figures 5E–H). Functionally, we found that PLAAT4 overexpression restored the reduced expression of E-cadherin and increased the expression of cyclin D1, vimentin, and MMP1 in the BCL6-overexpressing SKOV3 and COV504 cells (Figures 5I,J).

### 3.6 BCL6 promotes HGSOC growth and metastasis by PLAAT4 *in vivo*

**FIGURE 6:**
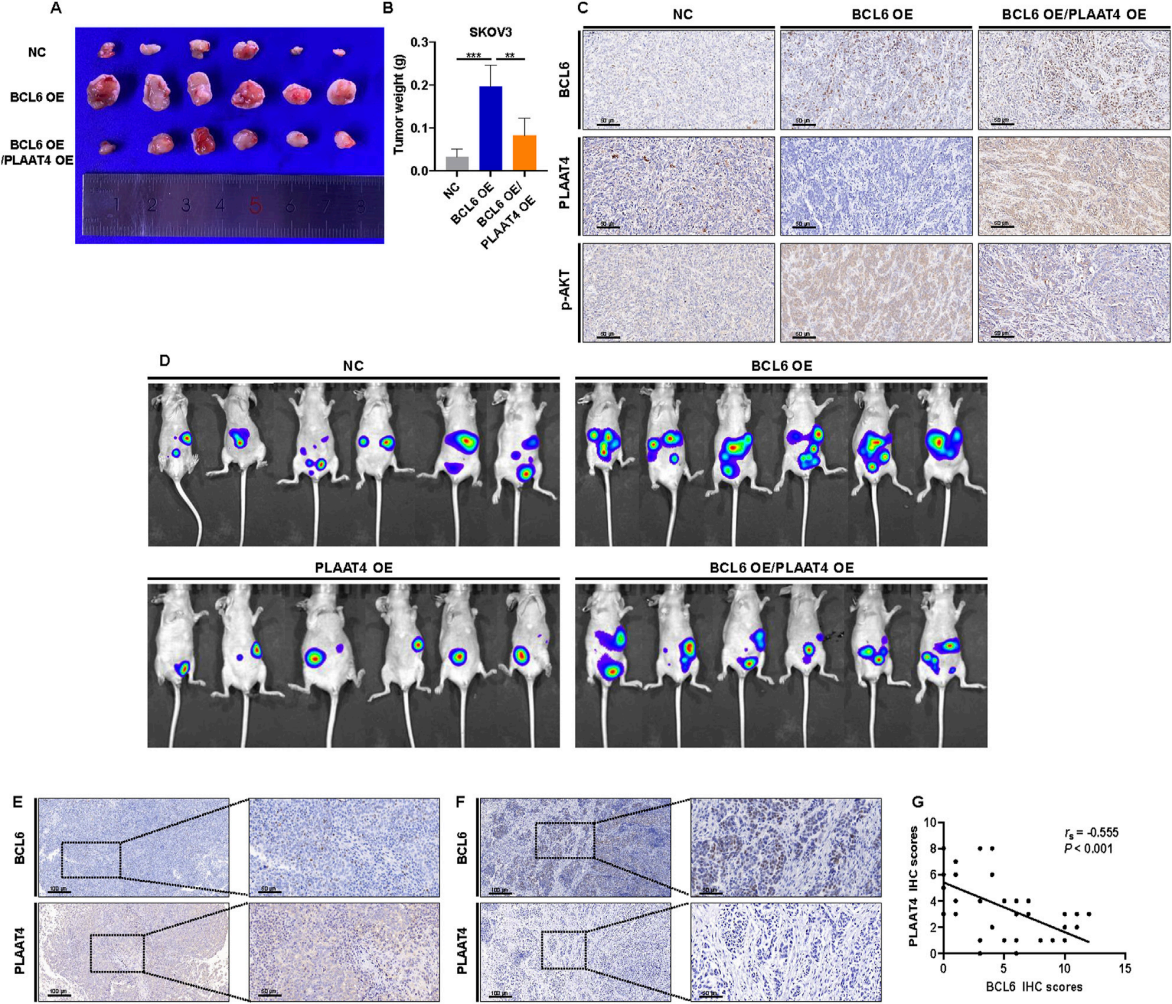
BCL6 promotes HGSOC growth and metastasis via PLAAT4 *in vivo*. **(A,B)** BCL6-overexpressing SKOV3 cells, with or without PLAAT4 re-expression, were subcutaneously injected into nude mice. Images **(A)** and weights **(B)** of the excised xenografts recovered at 21 days. Data represent mean ± SD, n = 6 mice per group. **(C)** Representative IHC staining of BCL6, PLAAT4 and p-AKT in tumors derived from nude mice. **(D)** Bioluminescence imaging at 28 days after intraperitoneal injection using IVIS Imaging System. **(E,F)** HGSOC tissues were analyzed in parallel for the expression of BCL6 and PLAAT4 via immunohistochemical (IHC) staining. Representative staining of cases with low and high expression of BCL6, respectively **(E,F)** and quantification of IHC staining as immunoreactive scores (IRS) **(G)**. *P < 0.05, **P < 0.01, ***P < 0.001, ns, not significant.

To confirm whether the BCL6-PLAAT4 axis plays the same role *in vivo* as that *in vitro*, we established a subcutaneous transplantation tumor and abdominal metastasis model of the SKOV3 cells in nude mice following replenishment of either BCL6 gene or co-replenishment of both BCL6 and PLAAT4. Following subcutaneous implantation in nude mice, BL6-overexpressing cells showed a higher rate of tumor growth than NS cells. Meanwhile, cells that co-overexpressed BCL6 and PLAAT4 showed similar rates of tumor growth as NS cells (Figures 6A,B). IHC analysis of the respective tumor samples showed efficient suppression of PLAAT4 upon overexpression of BCL6, while the expression of p-AKT was significantly increased. However, co-overexpression of PLAAT4 markedly restored p-AKT expression (Figure 6C). Furthermore, the same result was observed in the tumors formed in the abdominal metastasis model, where it was evident that abdominal metastasis was significantly increased after overexpression of BCL6. Conversely, in cells transfected with PLAAT4 alone or in combination, the opposite effects were observed (Figure 6D). Finally, we considered the clinical relevance of the BCL6 and PLAAT4 proteins in human HGSOC. If the mechanism was relevant in this setting, the expression of BCL6 would be negatively correlated with that of PLAAT4. To explore this notion, we used immunohistochemistry based on serial sections to measure the expression of BCL6 and PLAAT4 in HGSOC tissue. This analysis revealed a clear trend wherein HGSOC tissues with low BCL6 expression had higher PLAAT4 expression, while the reverse was true for HGSOC tissues with high BCL6 expression (Figures 6E,F). Quantitative assessment of staining also revealed a negative relationship between the expression of the BCL6 and PLAAT4 proteins (P < 0.001, Figure 6G).

## 4 Discussion

BCL6, a member of the BTB family of transcriptional repressors, promotes cancer progression (Fernando et al., 2019; Li et al., 2022; Zeng et al., 2023; Takamura et al., 2023) through participation in various cellular signal transduction pathways—including those involving proteins such as p53 (Hatzi and Melnick, 2014) —by binding to target gene promoters to suppress transcriptional programs (Cardenas et al., 2017). While previous studies have predominantly identified BCL6 as a hematopoietic tumor-specific oncogene (Kawabata et al., 2021), intriguing emerging data have also linked BCL6 to the regulation of the progression of solid tumors, such as lung cancer (Li et al., 2022), breast cancer (Yu et al., 2015), and ovarian cancer. Consistent with these findings, our clinical pathological analysis revealed that BCL6 is upregulated in HGSOC tissues relative to normal fallopian tube tissues. Moreover, BCL6 overexpression was found to be positively correlated with advanced tumor stage and poor prognosis in patients with HGSOC. Additionally, we observed that BCL6 enhanced HGSOC cell proliferation, invasion, and migration, whereas BCL6 knockout significantly reduced tumor growth in nude mice.

RNA-seq analysis of SKOV3 cells overexpressing BCL6 revealed that BCL6 activates various signaling cascades, with the expression levels of the PI3K-AKT pathway being the highest. The PI3K-AKT pathway is commonly activated in malignant cells and is associated with processes such as cell proliferation, metastasis, and chemotherapeutic resistance across a spectrum of malignancies, including endometrial (Wik et al., 2013), gastric (Huang et al., 2017), breast (Luo et al., 2018), and lung (Jin et al., 2022) cancers. Additionally, recent reports have indicated that BCL6 suppresses FOXO3 activity through the activation of the PI3K‒AKT signaling pathway in urinary bladder urothelial carcinoma (Wu et al., 2020). Consistent with these observations, we found that BCL6 enhances PI3K-AKT signaling in HGSOC cells. To our knowledge, this study is the first to provide evidence that BCL6 regulates cell proliferation, invasion, and migration via the PI3K-AKT signaling pathway through the use of PI3K-AKT inhibitors. The PI3K-AKT pathway serves as a primary signaling pathway in various cancer types and can participate in tumorigenesis through multiple effector mechanisms (Guo et al., 2024; Ke et al., 2024; Zhang et al., 2024).

To further elucidate the mechanism by which BCL6 regulates the PI3K-AKT pathway, we observed a negative correlation between BCL6 and *PLAAT4* expression levels. The activation of BCL6 can inhibit *PLAAT4* transcription by binding to its promoter region, resulting in reduced *PLAAT4* expression, which in turn suppresses the activation of the PI3K–AKT pathway. PLAAT4, also known as retinoic acid receptor responder 3 (*RARRES3*), retinoid-inducible gene 1 (*RIG1*), or tazarotene-induced gene 3 (*TIG3*), is classified as a class II tumor suppressor and is a member of the HREV107 protein family (DiSepio et al., 1998). Its expression is widespread in human cells. As PLAAT4 is a target gene of the nuclear transcription factors p53 and IRF1, *its* transcriptional activation can be increased by various stimuli in human cells, with roles in anti-oncogenesis and pathogen resistance (Hsu et al., 2012; Matsumoto et al., 2023). Consistent with these findings, our study demonstrated that PLAAT4 could attenuate the proliferation, invasion, and migration of HGSOC cells.

**FIGURE 7:**
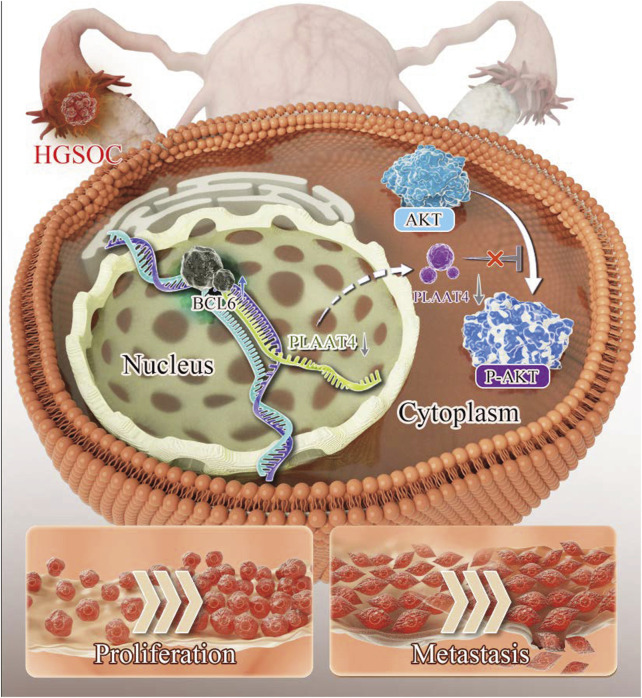
Schematic diagram showing how BCL6 regulates malignant behaviors in HGSOC through the transcriptional control of *PLAAT4*.

As a tumor suppressor, PLAAT4 can inhibit tumor progression through various pathways, with the PI3K-AKT pathway being one of the most significant mechanisms. For example, IFN-gamma induces RIG1 expression, leading to reduced phosphorylation of AKT while still retaining partial tumor-suppressor functions (Ou et al., 2008). Additionally, activation of the Wnt/β-catenin and PI3K-AKT pathways plays a crucial role in PLAAT4-silenced breast cancer cells (Hsu et al., 2015). Consistent with these findings, our research revealed that the activation of BCL6 could suppress the transcription of *PLAAT4* by binding to its promoter region, thereby contributing to the activation of the PI3K–AKT pathway (Figure 7).

While this study provides mechanistic insights into BCL6-driven HGSOC progression, several limitations warrant consideration. Our *in vitro* findings rely primarily on two established cell lines (SKOV3 and COV504). Although the results are representative of high-grade serous ovarian cancer, future studies should validate key findings in a broader panel of cell lines, organoids, or primary cells to enhance their generalizability. Furthermore, our *in vivo* models utilized subcutaneous and intraperitoneal xenografts in nude mice. While these models demonstrate tumorigenicity and response to intervention, they do not completely recapitulate the complex human tumor microenvironment, particularly the role of adaptive immunity.

## 5 Conclusion

In summary, clinical analyses reveal that BCL6 expression is significantly elevated in HGSOC tissues relative to normal tissues, whereas PLAAT4 expression is reduced. Moreover, high BCL6 and low PLAAT4 expression are associated with poor prognosis in patients with HGSOC. Mechanistically, we found that BCL6 inhibited *PLAAT4* transcription by binding to its promoter region, resulting in reduced PLAAT4 expression, further promoting AKT phosphorylation. Collectively, these findings elucidate the pivotal role of the BCL6-PLAAT4-AKT axis in HGSOC progression, establishing a molecular framework for targeting this pathway as a potential therapeutic strategy against HGSOC.

## Data Availability

The datasets presented in this study can be found in online repositories. The names of the repository/repositories and accession number(s) can be found in the article/Supplementary Material.

## Abbreviations

ATCC, American Type Culture Collection; BCL6, B-cell lymphoma 6; BTB, Broad-complex, tramtrack, and bric-a-brac; DEGs, Differentially expressed genes; EdU, 5 Ethynyl 2 deoxyuridine; GC, Germinal centers; FBS, Fetal bovine serum; GSEA, Gene set enrichment analysis; HGSOC, High-grade serous ovarian cancer; OS, Overall survival; PLAAT4, Phospholipase A and acyltransferase 4; RD2, Repressor domain; USTC, University of Science and Technology of China.
