# Supplementary material for: BCL6 promotes the progression of high-grade serous ovarian cancer cells by inhibiting PLAAT4
Source: Front Pharmacol. 2025 Jul 24;16:1634995. doi: 10.3389/fphar.2025.1634995 (PMC12328348; doi:10.3389/fphar.2025.1634995)
Supplement: Supplementary file 1 [file Supplementaryfile1.docx]

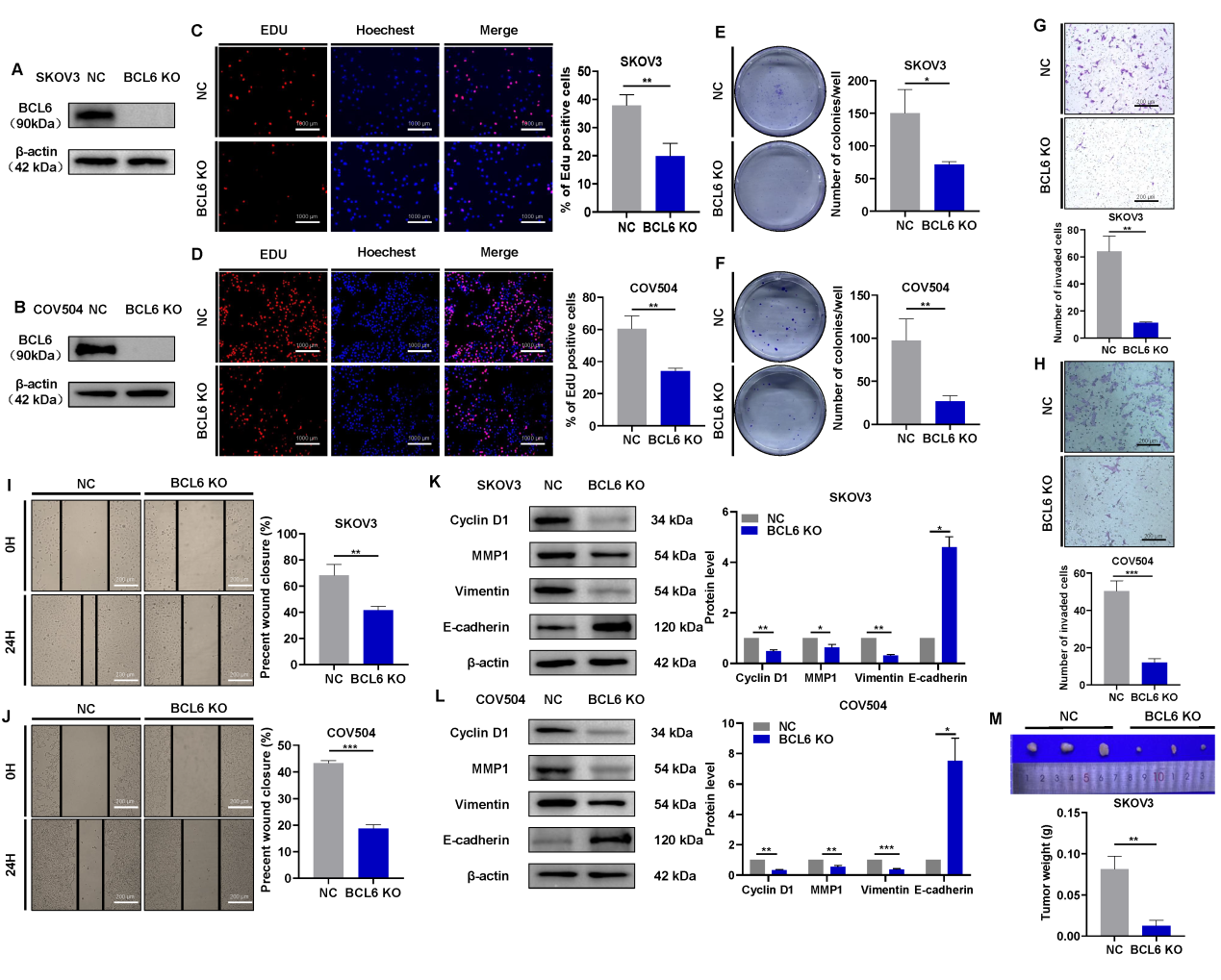


Supplementary Fig. 1 Knocking out BCL6 inhibits malignant behaviors in HGSOC cells. A B Western blot analysis of the HGSOC cell lines SKOV3 (A) and COV504 (B) after transfection with sgRNA to knockout BCL6. β-actin was used as a loading control. C Assessment of cell proliferation in the cells from A measured by EdU assays (scale bar = 1000 µm). D Assessment of cell proliferation in the cells from B measured by EdU assays (scale bar=1000 µm). E Assessment of the colony formation ability of the cells from A, as measured by colony formation assays. F Assessment of the colony formation ability of the cells from B measured via colony formation assays. G Assessment of migration in the cells from A measured by wound healing assays (scale bar=200 µm). H Assessment of migration in the cells from B measured by wound healing assays (scale bar=200 µm). I Assessment of invasion in the cells from A measured by transwell assays (scale bar=200 µm). J Assessment of the invasiveness of cells from B measured by transwell assays (scale bar=200 µm). K L Western blotting analysis of cyclin D1, vimentin, E-cadherin, and MMP1 in total cell lysates after knockout of BCL6 in SKOV3 and COV504 cells. M SKOV3 cells without (sg-NC) or with knockout of BCL6 (sg-BCL6) were subcutaneously inoculated into nude mice. Images and weights of the excised xenografts recovered at 21 days. Data represent mean ± SD, The data are presented as the means ± SDs; two-tailed student’s t test, *P <0.05, **P < 0.01, ***P < 0.001, ns: not significant.


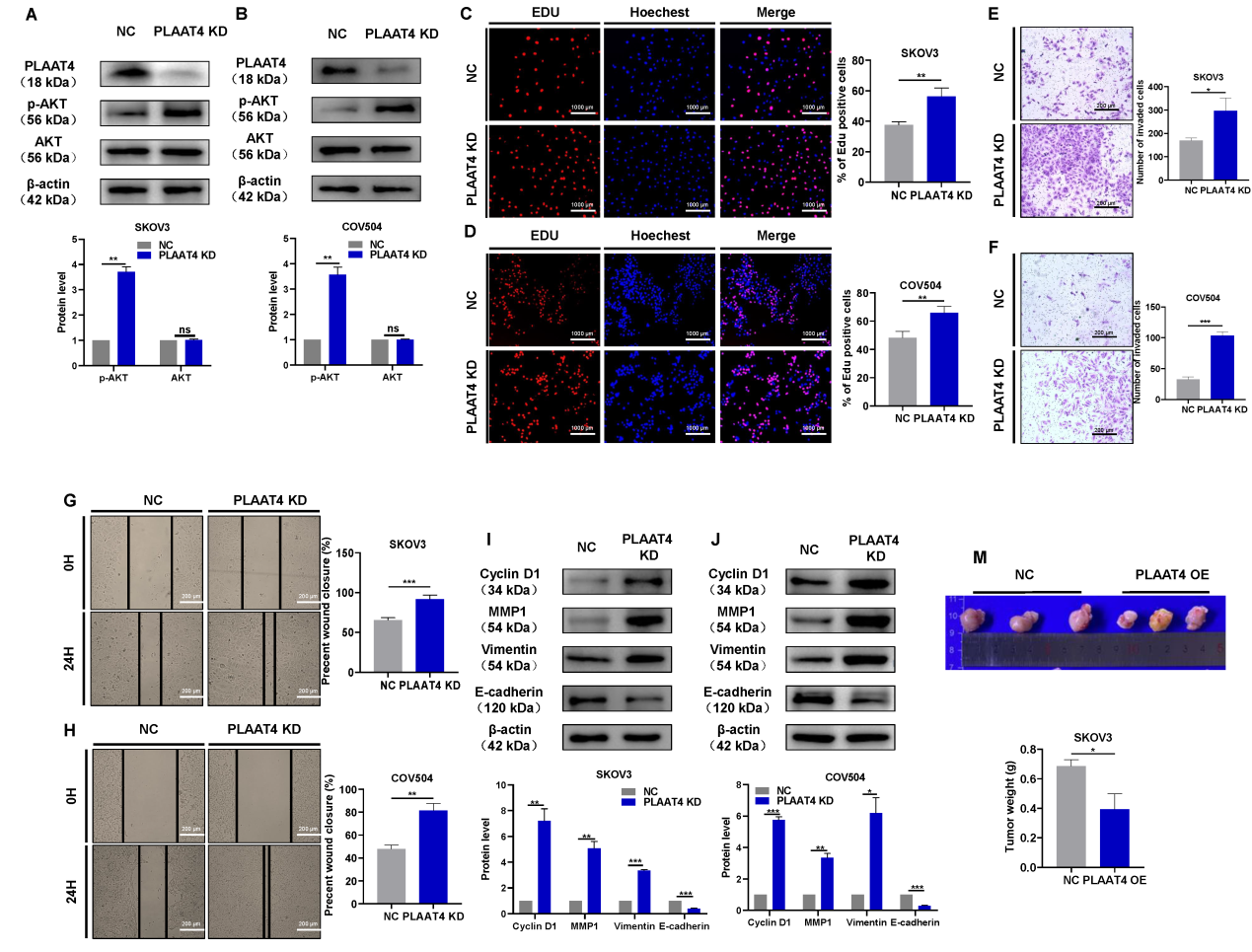


Supplementary Fig. 2 Reducing PLAAT4 levels can promote HGSOC cell malignant behaviors. A B Western blot analysis of AKT and p-AKT in total cell lysates after PLAAT4 was knocked down in SKOV3 and COV504 cells. C Assessment of proliferation in SKOV3 cells via EdU incorporation assays (scale bar=1000 µm). D Assessment of COV504 proliferation measured via EdU assays (scale bar=1000 µm). E Assessment of the invasiveness of SKOV3 cells via transwell assays (scale bar=200 µm). F Assessment of the invasiveness of COV504 cells via transwell assays (scale bar=200 µm). G Assessment of migration in SKOV3 cells via wound-healing assays (scale bar=200 µm). H Assessment of COV504 migration in wound-healing assays (scale bar=200 µm). I J Western blot analysis of cyclin D1, vimentin, E-cadherin, and MMP1 in total cell lysates after knockdown of PLAAT4 in SKOV3 and COV504 cells. M SKOV3 cells without (pCMV-NC) or with overexpression of PLAAT4 (pCMV-PLAAT4) were subcutaneously inoculated into nude mice. Images and weights of the excised xenografts recovered at 21 days. Data represent mean ± SD, The data are presented as the means ± SDs; two-tailed student’s t test, *P <0.05, **P < 0.01, ***P < 0.001, ns: not significant.
